# The technology of polychrome glazed ceramics in *Ifriqiya*: new data from the site of Chimtou

**DOI:** 10.1007/s12520-024-01974-x

**Published:** 2024-03-23

**Authors:** V. Occari, H. Möller, C. Fenwick, P. Quinn, I. C. Freestone, M. Chaouali, P. von Rummel

**Affiliations:** 1 UCL Institute of Archaeology, London, WC1H 0PY UK; 2 Deutsches Archäologisches Institut, Berlin, Germany; 3 Institut National de Patrimoine, Tunis, Tunisia

**Keywords:** Glaze technology, Medieval Tunisia, Recycling, Tin opacification, Cross-craft interaction, Polychrome glazed ceramics

## Abstract

*Ifriqiya* (roughly Tunisia and eastern Algeria) is believed to have played a significant role in the diffusion of ceramic glazed technologies into other regions of the Western Mediterranean. However, due to limited analysis on North African glazed ceramics, its role in technology transfer remains poorly understood. This paper uses SEM–EDS and petrographic analyses to understand the technology employed in the production of Tunisian ceramics through the study of 30 polychrome glazed ceramics from a medieval settlement at the site of Chimtou (ancient Simitthus), Tunisia, dated to the late ninth-twelfth century. The results show that these are lead-rich glazes with varying contents of alkalis, coloured with copper, iron and manganese oxide and applied over a calcareous body. Opaque glazes were obtained using cassiterite crystals as opacifier or by adding crushed quartz. The use of lead stannate as a colourant and opacifier in one light yellow glaze raises questions about the mechanisms of introduction of tin opacification technology in North Africa. Scrap metal seems to have been used as a source of lead for the glazes; while iron slag was probably used as a source of iron to colour the glaze in one sample, pointing to a cross-craft interaction between glazemaking and metallurgy.

**Supplementary information:**

The online version contains supplementary material available at 10.1007/s12520-024-01974-x.

## Introduction

The glazed technology “revolution” in the Islamic world in the late eighth century transformed pottery production, consumption and its everyday use. Once thought to originate in 9th-century Abbasid Iraq (Mason and Tite 1997; Sarre 1925; Tite and Wood 2005), Islamic fine glazed ceramics opacified with tin are now believed to have been first produced in the late seventh -eighth century in Egypt and then Syria with the emergence respectively of the so-called Coptic Glazed Wares (CGW) and Yellow Glaze Family (YGF), decorated with a brown or black paint under a yellow-amber glaze or with bands of green, yellow, brown, white and colourless glazes (Matin et al. 2018; Scanlon 1998; Tite et al. 2015; Watson 2014, 1999). From there, glaze technology appears to have been adopted across the Islamic world via multiple routes (Matin et al. 2018; Salinas et al. 2022a; Tite et al. 2015; Watson 2014).

In *Ifrīqiya* (roughly Tunisia and eastern Algeria)*,* polychrome glazed ceramics were produced by the late ninth century, when yellow glazes were applied over a painted background in the so-called “Jaune de Raqqada” wares in the Kairouan region and distributed across North Africa (Ben Amara et al. 2001; Gragueb Chatti 2017). As such, glazed ceramics are one of the primary markers for medieval occupation in North Africa (Fenwick 2020). Polychrome glazed wares with a white opaque background, which recall the contemporary tin-opacified white Iraqi wares (Matin 2019; Tite et al. 2015; Wood et al. 2007), have been found in contexts dated to the tenth century; however in Tunisia this seems to have been initially obtained using crushed quartz, or by whitening bleaching the ceramic surface through the addition of salt water to the ceramic paste (Salinas et al. 2020). The production of glazed wares was a notable departure from previous North African ceramic traditions and entailed both the adoption of glazing technologies and the emergence of new shapes and decorative styles (Gragueb Chatti et al. 2019).

During the Fatimid-Zirid period new glazes wares were produced probably in different workshops, one of which was Sabra al-Mansuriyya (tenth-eleventh century) just outside Kairouan (Ben Amara et al. 2005). Chemical analyses of the wares from both sites by Ben Amara et al. (2001, 2005) indicated that these were decorated using a transparent high lead glaze coloured with iron and that a copper and manganese-based pigment was used respectively for the green and brown decorations, with a repertoire of mainly geometric, epigraphic but also zoomorphic and anthropomorphic décor (Cressier and Fentress 2011; Gragueb Chatti 2017). Although they identified similar techniques and raw materials in the two groups of wares (i.e. the use of calcareous clay, high lead glaze and same type of colourants), the method of application of the glazing mixture differed, with the Raqqada wares manufactured in a double firing, and the Sabra al-Mansuriyya ones in a single firing. Scholars have also examined the introduction of tin-glaze technology and Salinas et al. (2019a) have recently argued that tin opacification reached Ifriqiya only *after* the Fatimid conquest of Egypt (969 CE) -where this technology had been in use since the eighth century (Tite et al. 2015).

Aghlabid *Ifriqiya* is generally believed to have played a prominent role in the diffusion of glazed technology to north-western Africa, Iberia and Sicily but the nature and direction of this technological connection is still debated (Salinas et al. 2019a; Salinas et al. 2022b; Waksman et al. 2015). Polychrome glazed ceramics with comparable designs, patterns and shapes are found at other 9th to 11th century sites in Tunisia, which are largely assumed to have been produced in the Kairouan region and then diffused, as well as in Algeria, Sicily and Libya, which have been the focus of recent chemical and petrographic studies (Mokrani 1997; Sacco 2017; Salinas et al. 2019b; Waksman et al. 2015). A recent paper by Djellid et al. (2023) underscores the clear technological connection between an early group of polychrome glazed ceramics from Tahert (Algeria, ninth century) and Tunisian ceramics. The development of glazed technology in Palermo (Sicily) in the late ninth century is traditionally thought to have been the result of the movement of artisans from Aghlabid *Ifriqiya* to Aghlabid Sicily. There are strong technological and stylistic similarities between the two productions, and they share the same typo-stylistic evolution during the late ninth-eleventh centuries (Ardizzone et al. 2017; Capelli et al 2020; Sacco 2017). On the other hand, it has been recently suggested that glazed wares developed independently in al-Andalus through technological transfer from a new autochthonous lead-based glass technology to ceramic glazing techniques (Salinas et al. 2019b; Salinas et al. 2022a). However, due to limited analyses of Tunisian glazed ceramics, the transmission of new technologies in the central Mediterranean remains poorly understood.

We still know very little about how and where Tunisian glazed ceramics were manufactured, whether there were multiple production centres, which raw materials were used and how Tunisian productions relate to other Mediterranean glazemaking traditions. This paper provides new insights to these issues through the study of a glazed ceramic assemblage dated to the ninth-twelfth century from the archaeological site of Chimtou (ancient Simitthus), in the Medjerda Valley, northern Tunisia. Compositional data on the glazes provide evidence not only about the technology and recipes employed but also – when combined with the chemical and petrographic data on the ceramic body – on the organisation of production and specialised workshops (Andrews 1997; Klesner et al. 2021; Ting et al. 2021; Tite et al. 2008). Scanning electron microscopy energy-dispersive spectrometry (SEM–EDS) and thin-section petrography have been used to identify the technology employed to manufacture the glazed ceramics, including the type of raw materials used, the preparation method of the glaze and the body, the method of application of the glaze, and the possible provenance of the ceramics. The data obtained are compared with those of published contemporary 9th-12th c. Tunisian and Algerian assemblages to identify commonalities in the technological processes used.

## Materials and methods

### The archaeological site of Chimtou

**Fig. 1:**
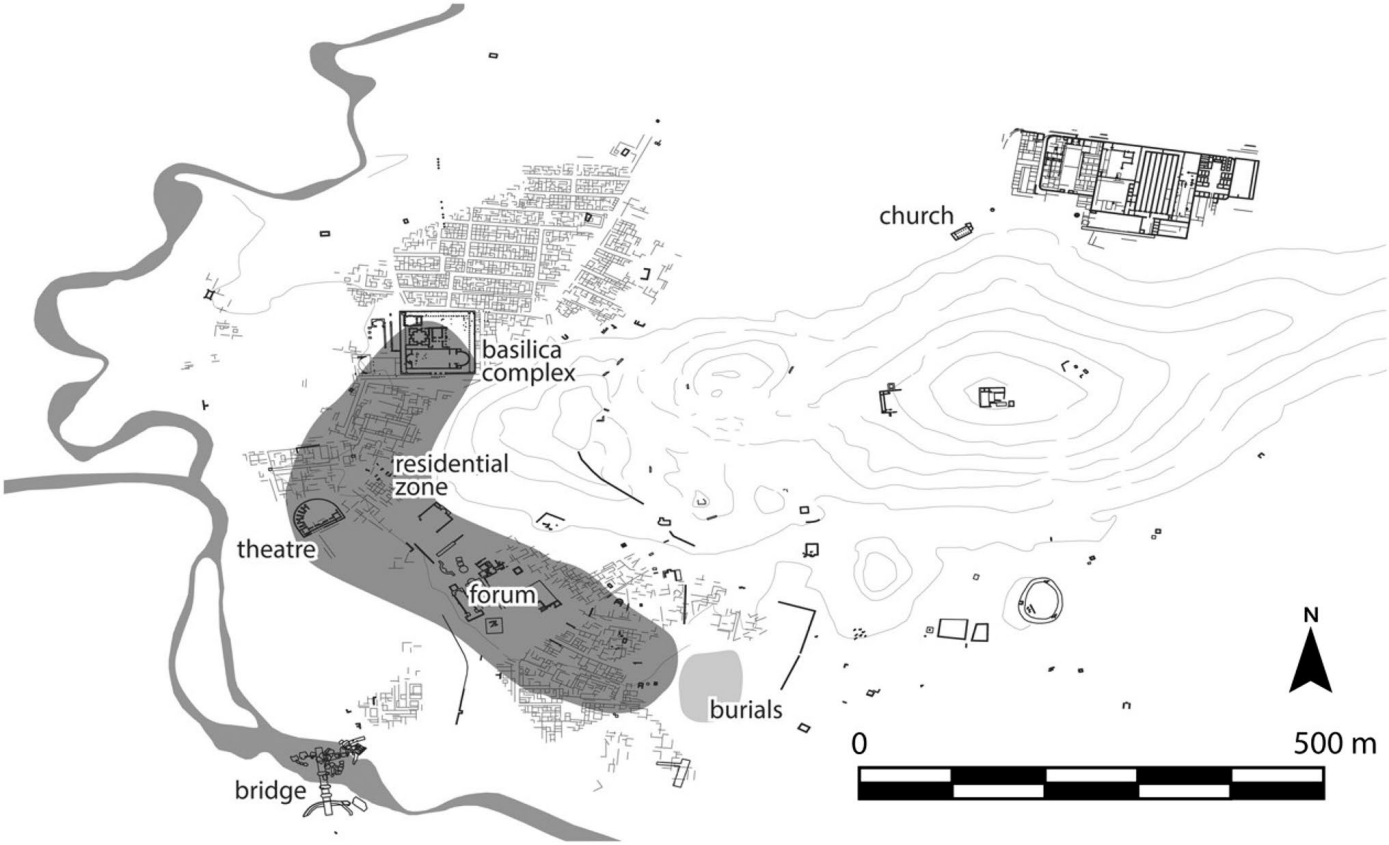
Plan of Chimtou showing location of medieval settlement and key places named in the text

The modern town of Chimtou (ancient Simitthus, Tunisia) (36° 29′ 31″ N, 8° 34′ 34″ E) was an important Numidian and then Roman town of c. 80 hectares in size and famous for its quarries under Roman imperial control which exported yellow marble across the Mediterranean (Ardeleanu et al. 2019; Beck 2024; Rakob 1993). Medieval activity has been identified by archaeologists since the late 1890s and it seems there was a sizeable settlement here in the ninth-twelfth centuries, though significantly smaller than the Roman city (Fig. 1, Fenwick et al. 2022; von Rummel and Möller 2019).

Excavations by the DAI and INP in the late 1970s-1980s and 2010s and the DAI (Deutsches Archäologisches Institut, Berlin), INP (Institut National de Patrimoine, Tunis) and UCL in 2022 have uncovered stratified sequences of medieval settlement north-east of the forum and in the north-west of the city in a temple/ basilica complex. Excavations suggest a rupture in occupation in these zones at least: both the late antique forum and temple/ basilica complex were abandoned by the seventh century and covered with a deep layer of deposits onto which new one-storied structures were built and occupied between the ninth and twelfth centuries (Khanoussi and von Rummel 2012: 184–92; von Rummel and Möller 2019: 178–95). The forum area was given over to small flat-roofed rectangular, one-storied buildings built in rubble and spolia with courtyards or exterior open spaces with storage silos. Inside these buildings, several occupation phases correspond with a succession of floors (House P, cf. von Rummel and Möller 2019: 192, Fig. 9). The assemblage is chronologically very mixed. Small, fragmented early Roman sherds, mainly tableware and lamps, are mixed with the equally poorly preserved medieval sherds, including kitchen and table ware with the most recent vessels dating to the 11th/twelfth century. A kiln excavated in the forum area in the early twentieth century and associated with a tenth century coin hoard (Toutain 1893: 467–8) suggests medieval ceramic production at the site, though presumably of unglazed wares. The excavations in the temple / basilica complex show a very similar picture but unfortunately the 1980s excavations did not document the medieval features. The most recent excavations confirm that here too, between the ninth -twelfth centuries, the walled complex was filled with rectangular, flat-roofed single-storey buildings and storage silos. After the abandonment of the medieval settlement, the silos were used as rubbish pits: these contain mainly 6th—mid seventh century pottery and only a few medieval sherds.

### The glazed ceramics from Chimtou

The 30 samples selected are representative of the medieval ceramics excavated at Chimtou to date. The medieval ceramics were produced between the late 9th to the mid-twelfth century, however, it is currently difficult to establish a strong typo-chronology due to the high levels of residuality, the small-scale excavation of medieval contexts and the limited publications of stratified medieval ceramics in Tunisia. Nevertheless, the repertoire gives a first impression of the household inventory of medieval houses in Chimtou. Glazed ware is poorly represented, and the assemblage consists mainly of plain wares, which include handmade kitchen ware such as so-called Maâjnas and jars as well as wheel-thrown bowls and filter jugs (Fenwick et al. 2022; von Rummel and Möller 2019). While the handmade and wheel-thrown cookware was produced locally or in the immediate region and there are very few intra-regional imported wares, the limited glazed ware does suggest trade with central or north-eastern Tunisia. Imports from as far as Sicily are known among the transport amphorae (amphora D’Angelo E1/2: Touihri 2016: 246, Fig. 5.6; von Rummel and Möller 2019: 209). The material can be roughly categorised chronologically and the amount of material from the 9th/tenth century is quantitatively far superior to that of the 11th/mid-twelfth century CE.

**Fig. 2:**
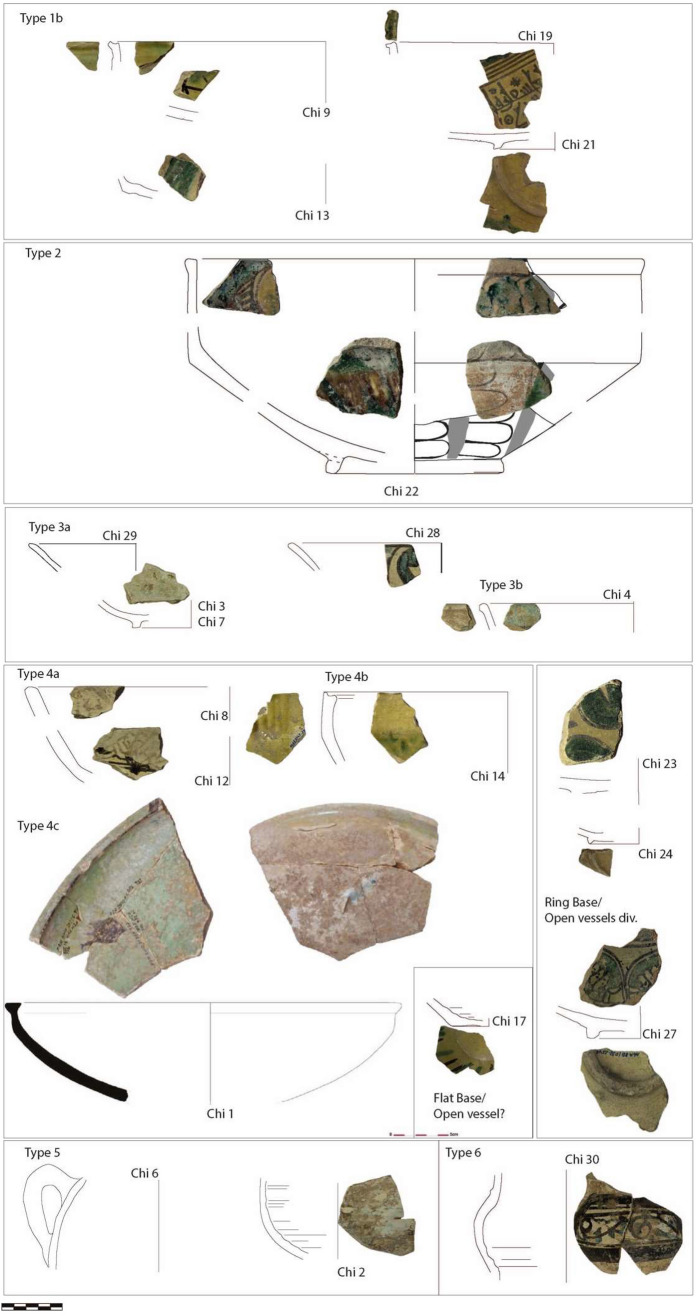
General types of the glazed wares from Chimtou

The typological range of glazed wares at Chimtou is limited to a few, mainly open, vessel-forms which were widely distributed across Africa and Sicily and are attested in almost all medieval Tunisian assemblages (Gragueb Chatti 2017). The glazed wares include cups and bowls in the distinctive yellow ceramics typical of Raqqada ('jaune de Raqqada') as well as small amounts of so-called Sabra al-Mansuriya ware. They can be preliminarily divided into six types (with sub-types), which are summarised in Fig. 2. Most common are carinated open bowl forms, as seen elsewhere (Louhichi 2010; Reynolds 2012; Daoualatli 1995:196), which are characterised by a rounded (type **1A**) (published in von Rummel and Möller 2019, 206: Fig. 17.57) or straight, sometimes slightly concave and pinched rim (type **1b**: Chi 9; 13; 19; 21) (Touihri 2016; Rossiter et al. 2012: 251). The diameters of the vessels are usually around 20 cm. They can be unglazed (von Rummel and Möller 2019: 206, Fig. 17.61), but usually bear a mustard-yellow lead glaze with some featuring geometric, and possibly also zoomorphic and floral patterns in green (copper) and brown (manganese) (Ben Amara et al. 2005; Daoulatli 1995; Gragueb Chatti 2013).

One selected sherd has epigraphic decoration (Chi 21) and resembles a bowl found in Raqqada dated to the first half of tenth century (Louhichi 2010: 60; Hamdi 2018: 239–241, Fig. 12.6A), however, in general, this vessel type and fabrics are typical of ninth and tenth century production. This group typologically also includes large deep carinated bowls, c. 30 cm in diameter with a thickened rim on the outside, decorated on the outside and inside with the same mustard-yellow lead glaze with some featuring geometric designs in green (copper) and brown (manganese) (type **2**: Chi 22; 31, see von Rummel and Möller 2019: 206, Fig. 17.55). Like the smaller examples (**1a** and **b**), they typically have a ring base (approximately 15 cm in diameter). One example (Chi 17) has a flat base and could belong to a pot (Louhichi 2010:46, Fig. 14). A similar piece was found in Utica (Salinas et al. 2022: 224, Fig. 2a.UT3). Other bowls or plates of similar diameter (type **3**) have an everted wall with ring base and rounded (**3a**) or thickened (**3b**) rim and occur mainly in greenish-turquoise (Chi 3; 4; 7; 28; 29). Rarer are larger (diameter ca. 30 cm) slightly carinated bowls (type **4**) with rounded rim (**4a:** Chi 8), slightly concave rim top (**4b:** Chi 14, Salinas et al. 2022: 224, Fig. 2b.UT2) or a smooth rim on top, thickened on the outside and inside of brown decoration and greenish glaze (**4c:** Chi 1). The latter was produced in the 11th/twelfth century (Vitelli 1981; discussed in Möller et al. 2012:33, Fig. 21.5). A handle fragment (type **5:** Chi 6) attached to a closed vessel and a body-sherd (Chi 2) probably also belongs to the same period (related to Louhichi 2010, 65).

In contrast to the open forms, there are very few glazed closed vessels. In the present sample set this includes only a jug/jar (pichet) (type **6:** Chi 30) unfortunately without preserved rim or base fragments. The attachment of a vertical handle that starts on the body and ends at the neck is typologically comparable to a vessel from Sabra al-Mansuriya (Daoualatli 1994:82–83, Fig. 37), although the fabric and white and brown glaze in geometric patterns differs (see Table 1). It might have a production date in the 12th or even thirteenth century.

### Methodology

Scanning electron microscopy energy-dispersive spectrometry (SEM–EDS) was used to determine the elemental composition of the glaze and of its associated body, the type of colourants employed and the method of application of the glaze. Sections of 30 samples were prepared in blocks polished to 1/4 micron, and carbon coated. The polished blocks were examined using a Carl Zeiss EVO25 SEM at the Wolfson Archaeological Science Laboratories at the Institute of Archaeology, UCL, fitted with an Oxford Instruments Aztec EDS analysis system. The SEM was operated at 20kV accelerating potential, with a deadtime of 35%-40% and a working distance set at 8.5 mm. The microstructures of the glaze and body were determined using backscattered electrons imaging (BSE) in order to distinguish between the different phases present, on the basis of their different mean atomic numbers. The compositions of both the glaze and the body were obtained by analysing at least three different areas. The area of analysis was about 50 × 25 μm on the glaze, while the areas of analysis of the ceramic body were about 150 × 75 μm at 200 × magnification. Sometimes the area analysed for the glaze was smaller to avoid weathered areas or areas close to the glaze-body interface. A selection of particles in the glaze such as opacifiers and neo-formed crystals were also analysed at higher magnification (between 600 × and 1000×). Calibration of the SEM–EDS results was obtained using the Aztec standardless factory calibration. Aztec uses a default database of standardisation for all elements for microanalysis, meaning that the user does not need to measure their own standards to produce quantitative EDS results. The spectra obtained were carefully inspected (i.e. peaks can be manually identified and confirmed) and data checked for quality control by comparison with reference materials, the analyses of which are given in the paper (Table S1). Presented values are estimated to be within 8% of the expected values for major elements and within 10% for minor elements except for SnO_2_, Sb_2_O_3_ and P_2_O_5_. The measurements were converted to oxide by stoichiometry and normalised to 100% due to the porosity of the samples and fluctuations in beam intensity. To evaluate accuracy and precision of the analysis, reference materials Corning Glass A, B, C and D were analysed, while beam current stability was checked by analysing cobalt at regular intervals (Supplementary Table S1). Oxides with concentrations lower than 0.1% are not reported as they were below the detection limits of the instrument.

In addition to the chemical analysis of the ceramic body and glaze, the ceramic paste was also investigated using thin-section petrography in order to study mineralogical and textural variations, to assess the presence of different fabrics and to determine the potential provenance of the samples. Standard 30 micron thin sections (Quinn 2022: 24–36) were prepared at the Wolfson Archaeological Science Laboratories and analysed using a LEICA DM EP Polarising microscope at magnification of 40–200 × and visually grouped into fabrics based upon the nature of their inclusions, clay matrix and voids (Quinn 2022: 89–97).

## Results

### Ceramic body

**Fig. 3:**
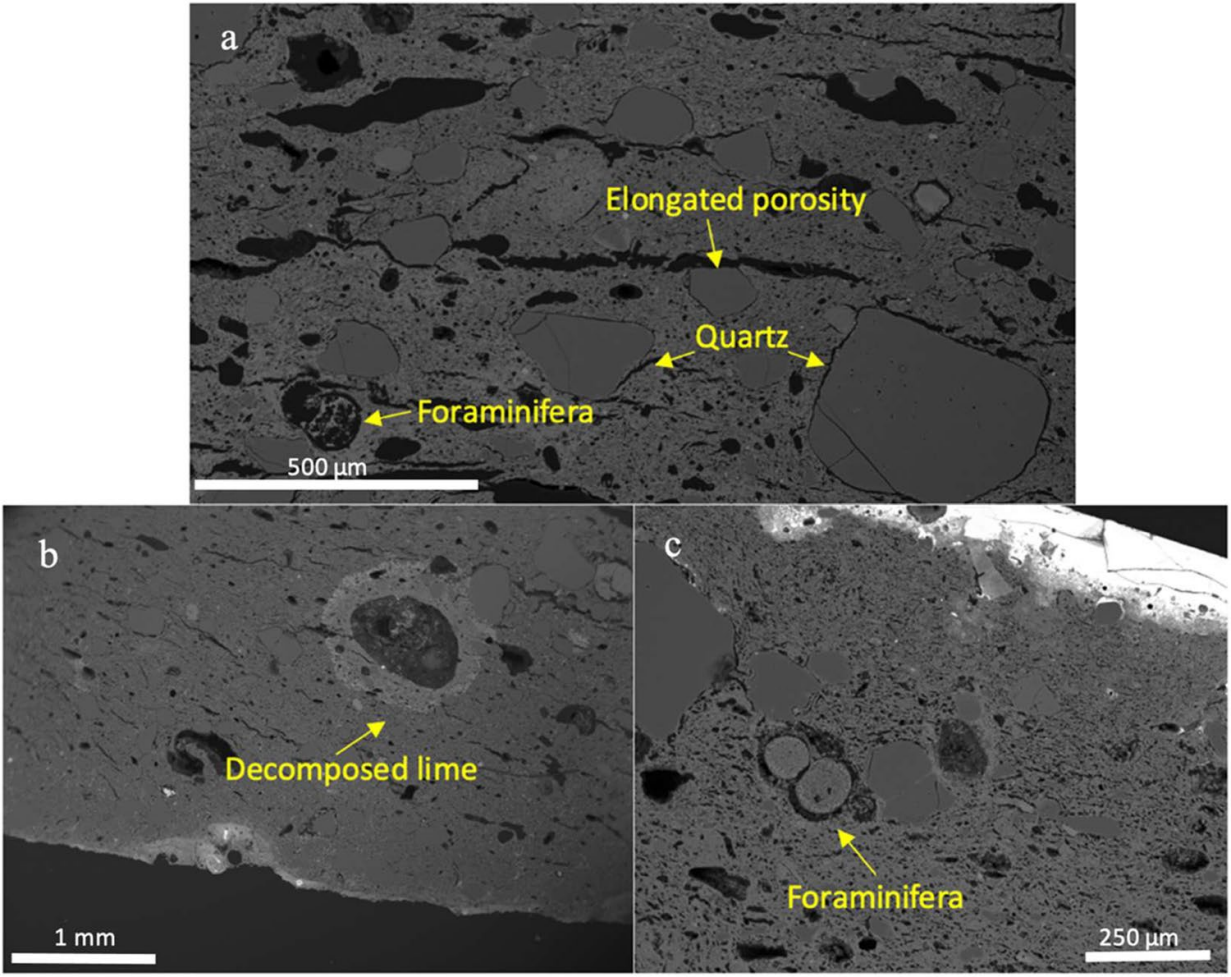
BSE images showing details of the microstructure of the ceramic bodies. **a**) Image showing characteristic elongated porosity, round quartz inclusions and microfossils inclusions (Chi 13). **b**) Image showing decomposed lime with white halos (Chi 21). **c**) Image showing detail of foraminifera microfossil (Chi 11)

SEM–EDS analysis of the ceramic bodies reveals that they are all made with calcareous clay, with variable CaO contents ranging from 12 to 33%, SiO_2_ from 28–64%, and a strong negative correlation between CaO and SiO_2_, which typically sum to 75%. It must be noted that the presence of secondary calcite precipitated from ground water during burial (Quinn 2022: 280–287) in many of the samples analysed (see below) can affect the total amount of CaO detected by the SEM–EDS, which might explain the relatively wide variation of CaO in the samples analysed. The bodies are also characterised by modest contents of Fe_2_O_3_ (4–7%), moderate Al_2_O_3_ contents (10–16.5%), low K_2_O (< 1.4%), significant levels of Na_2_O (1–2%) and MgO contents at around 2% (Tab. 1). Small concentrations of PbO are attributed to volatilisation during the glazing process. Under the SEM, the samples show sub-rounded to angular quartz grains as the main inclusions, elongate voids associated to the shrinkage of the body during drying and firing, as well as numerous voids after CaCO_3_ inclusions decomposed to CaO (Fig. 3). Carbonate inclusions generally start to break down at temperatures higher than 700–800 °C and their reaction with the surrounding clay matrix can leave the presence of “reaction rims”, which are clearly visible in the samples analysed and appear as white halos in Fig. 3b. (Drebushchak et al. 2005; Fabbri et al. 2014; Gliozzo 2020; Tschegg et al. 2009). The ceramic bodies also contain relics of foraminifera microfossils (Fig. 3c), rare feldspars and heavy minerals.

**Table 1:** Normalised chemical composition of the ceramic bodies determined by SEM–EDS

Sample	Fabric Group	Na_2_O	MgO	Al_2_O3	SiO_2_	K_2_O	CaO	TiO_2_	Fe_2_O_3_	PbO
Chi 1	3	1.2	1.4	12.4	50.9	0.34	26.5	0.7	5.3	0.7
Chi 2	2	1.3	1.8	13.8	48.5	0.8	25.3	0.7	5.8	2.0
Chi 3	3	1.2	3.1	13.8	57.9	0.6	15.1	0.8	5.5	1.9
Chi 4	3	1.3	1.9	14.4	59.0	1.2	13.7	0.7	5.3	2.5
Chi 5	2	1.0	1.5	12.2	52.5	1.0	24.9	0.6	5.5	0.7
Chi 6	2	0.9	1.8	12.5	45.6	1.6	28.9	0.6	5.6	2.5
Chi 7	3	2.0	3.0	14.3	54.4	0.9	15.6	0.8	5.9	3.1
Chi 8		1.9	1.9	15.3	59.8	1.6	13.0	0.8	5.3	0.5
Chi 9	1A	1.3	2.0	14.3	60.4	0.9	12.8	0.7	7.1	0.6
Chi 10	3	2.0	2.6	13.5	38.4	0.7	33.4	0.7	6.0	2.7
Chi 11	3	1.8	2.5	14.1	45.6	0.6	27.3	0.7	6.0	1.4
Chi 12		2.8	1.6	14.2	59.9	1.3	13.9	0.7	5.1	0.5
Chi 13	3	1.5	2.2	14.2	53.2	0.7	20.9	0.8	5.8	0.8
Chi 14	1B	1.4	2.5	15.4	45.1	0.9	24.8	0.9	7.5	1.4
Chi 15	1B	1.3	2.5	12.7	49.5	0.9	26.3	0.6	5.6	0.5
Chi 16	1B	0.8	2.0	10.8	56.5	0.7	22.6	0.6	4.9	1.2
Chi 17	1A	1.5	1.6	13.0	64.1	0.6	12.5	0.6	5.7	0.4
Chi 18	3	0.7	2.7	16.3	48.7	1.0	22.0	0.8	7.1	0.7
Chi 19	1A	0.9	2.1	15.6	55.1	0.8	17.5	0.6	6.1	1.3
Chi 20	1A	1.0	1.7	13.8	56.8	1.3	18.8	0.7	5.7	0.2
Chi 21	1A	1.0	1.7	13.8	56.8	1.3	18.8	0.7	5.7	0.2
Chi 22	3	1.7	2.8	14.0	49.9	0.7	24.9	0.6	4.3	1.0
Chi 23	1B	1.2	2.3	14.9	52.8	0.4	22.9	0.7	4.8	0.1
Chi 24	1B	1.4	2.3	15.0	50.1	0.7	23.0	0.8	5.9	0.9
Chi 25	1B	1.2	1.9	15.2	56.8	0.6	16.3	0.8	6.2	1.1
Chi 26	1A	0.7	2.6	13.6	48.5	1.4	25.0	0.7	5.7	1.8
Chi 27		1.7	2.0	17.2	50.6	1.5	17.1	0.8	6.0	3.1
Chi 28	2	1.9	2.6	16.5	43.9	0.9	25.6	0.7	6.6	1.2
Chi 29	3?	2.0	2.9	14.6	47.8	0.7	24.0	0.6	4.6	2.9
Chi 30	2	1.3	2.0	14.1	42.1	0.6	33.6	0.6	5.3	0.5

Although the samples appear quite homogenous in terms of their chemical composition, the ceramic paste has two main colours in hand specimen and thin section: the first has a reddish colour often with a lighter surface layer, while the second has a greenish colour throughout the whole thickness of the sherd. The presence of a lighter surface on the first fabric type is likely due to the use of salt water during the preparation of the calcareous paste, a tradition widely used in North Africa since the Punic period as well as in Mesopotamia and Pakistan (Bonifay et al. 2002–2003; Peacock 1984; Rye 1976). This type of discolouration takes place due to the combination of calcite and the sea water: the latter migrates to the surface during drying and promotes the reactivity between the lime and the clay minerals, leading to the formation of pyroxenes and a glassy phase which reduce the total content of iron available to form iron oxides (Molera et al. 1998). This process gives calcareous pastes a light green and then darker olive colour at temperatures between 1000 °C and 1100 °C (Maniatis et al. 1983; Matson 1971). It is therefore possible that the two fabric colours observed are a result of different firing temperatures rather than different clay sources. A whiter ceramic body would certainly have served as a more effective “canvas” for the subsequent painting and glazing of the ceramic, as a darker background colour of the ceramic bodies might have negatively interfered with the desired final colour of the glaze.

Petrographically all samples are characterised by a similar mineralogical composition, including grains of mainly rounded, subrounded to angular monocrystalline quartz, rare feldspar, iron-rich nodules and sandstone as well as abundant calcite inclusions which are very often decomposed. Microfossils are also visible in thin section, while several samples show the presence of secondary calcite. No other inclusions could be identified. While the nature of the inclusions is the same in all samples, some variation in the amount, distribution and size of inclusions can be observed which permit the subdivision of the samples into three main petrographic fabrics and one sub-fabric.

These contain some internal variation which could be the result of natural variation in the raw materials and paste preparation techniques.

**Fig. 4:**
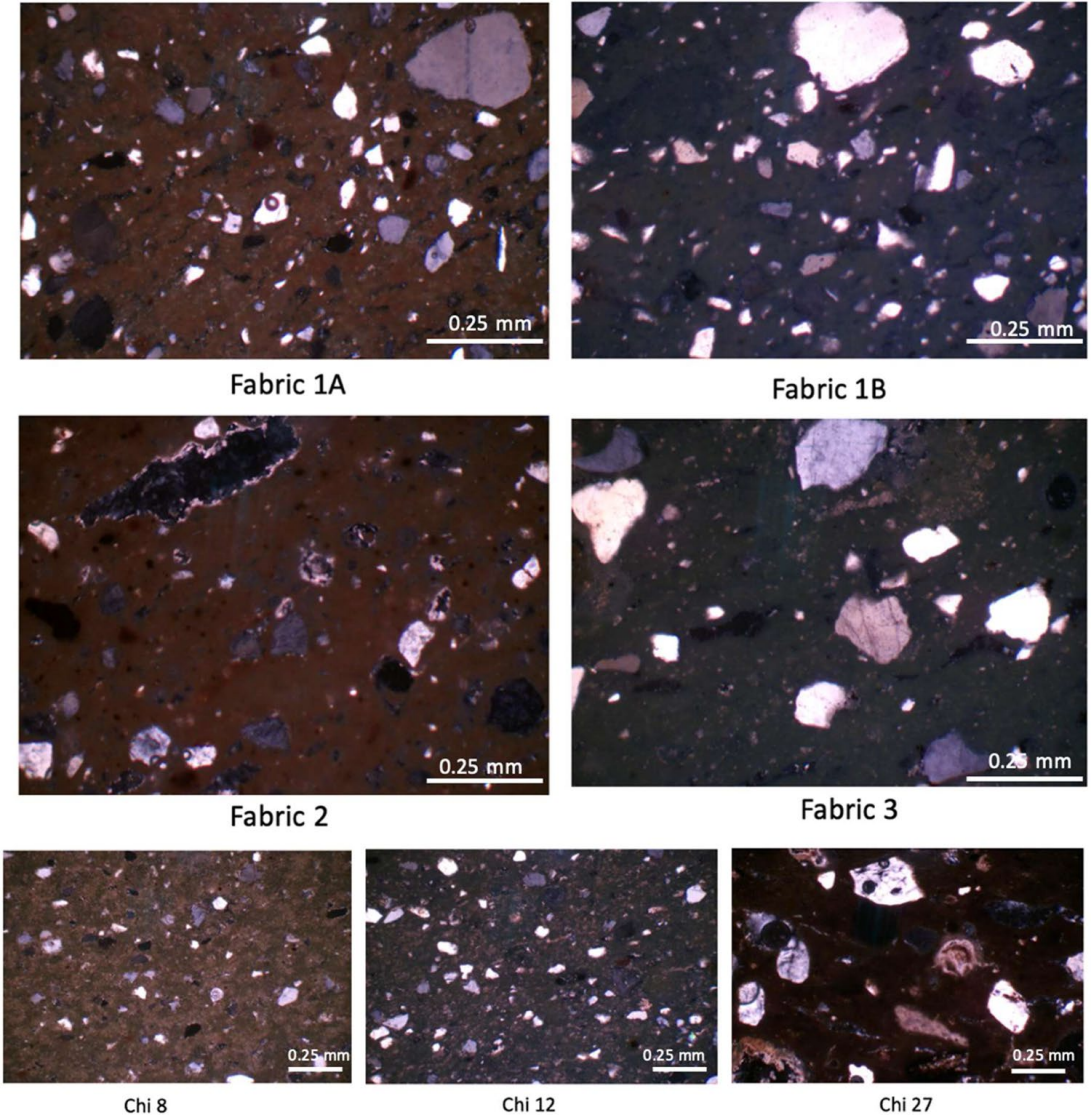
Photomicrographs showing the fabrics of the different petrographic groups identified and the three petrographic outliers  (Chi 8, Chi 12, Chi 27). All photomicrographs were taken in cross polarisation

**Petrographic Fabric 1A** is characterised by a reddish silty paste (silt inclusions size ranging from 0.01 mm to 0.06 mm), with an isotropic matrix with abundant sub-rounded to angular monocrystalline sand-grade quartz inclusions, with estimated abundance of inclusions of 30%. The inclusions are poorly sorted, ranging in size from 0.08 to 0.59 mm, and closed spaced. It cannot be excluded that medium sand was added as a temper to a silty clay matrix. Feldspars and iron-rich nodules are rare. Voids are predominantly elongated shrinkage cracks but round voids after decomposed calcite are also present (Fig. 4). Rare, preserved calcite inclusions (0.15–0.29 mm) can be observed in only few samples as well as microfossils. Chemically, the majority of Fabric 1A samples have silica contents at the high end of the range in the samples analysed (SiO2 > 55%). **Petrographic Fabric 1B** presents the same petrographic characteristic as Group 1, but it is characterised by an olive-green matrix, which is likely due to higher firing temperatures as noted above, perhaps in combination with its typically higher lime content. **Petrographic Fabric 2** is characterised by a reddish isotropic matrix, with a silty paste with less abundant and generally smaller inclusions than the other groups. The estimated abundance of inclusions is 10–15%, while the size varies between 0.10 and 0.17 mm, with rare coarser grains < 0.25 mm. The fabric also presents more voids, both rounded and vugh-like in shape, some of which might have been produced by the decomposition of calcite inclusions (Fig. 4). Fabric 2 bodies tend to have low silica and high lime concentrations relative to most other sherds (SiO2 < 52.5%, CaO > 24.9%). **Petrographic Fabric 3** is characterised by a cleaner matrix of a light green-olive colour. The inclusions are slightly better sorted (generally fine sand between 0.10 and 0.23 mm in size, with rare larger inclusions of medium sand grade < 0.64 mm) and are mostly sub-rounded in shape, with an estimated abundance of 10–15%. Few preserved calcite inclusions (< 0.47 mm) are also present. As in the case of fabric 1, it is possible that sand was added as temper to a purer matrix (Fig. 4). Group 3 is the largest identified (10 samples) and more-or-less spans the complete range of SiO_2_ and CaO contents.

Three sherds have unique petrographic compositions and are not related to the three main fabrics (Fig. 4). Samples Chi 8 and Chi 12 present a cleaner matrix with smaller and better sorted inclusions compared to the other samples. Inclusions are slightly more abundant and more closely spaced in sample Chi 12. The finer matrix with no sand-grade inclusions and with little silt grade inclusions might suggest that the clay paste could have been refined by settling or levigation by the potters for the manufacture of these ceramics (Quinn 2022: 216). Sample Chi 27 shows a paste relatively rich in large quartz inclusions, sub-rounded to angular in shape, associated with numerous large inclusions of rounded, rhombic and elongate fragments of micritic limestone and few foraminifera microfossils (Fig. 4). In terms of chemistry the three outliers have relatively high K_2_O and low CaO contents.

To summarise, a series of petrographic fabrics have been identified, separated mainly on the basis of their textural characteristics. In terms of their oxide compositions, they are closely related, but some support for the petrographic categories exists in the chemical composition of the paste, particularly for Fabrics 1B and 2, as well as the outliers. Given the relatively small sample size and anticipated imprecisions of the SEM–EDS analysis of tempered ceramic bodies, these correspondences are encouraging and may reflect the activities of different kilns.

### Glazes

The compositional data indicate that there is no apparent link between glaze decoration (i.e. colour, design) and body fabric type, implying that different workshops made ceramics with wide range of decorations. However, the poor preservation of some of the glazes sometimes hampers the interpretation of the type of colour and decoration present.

**Fig. 5:**
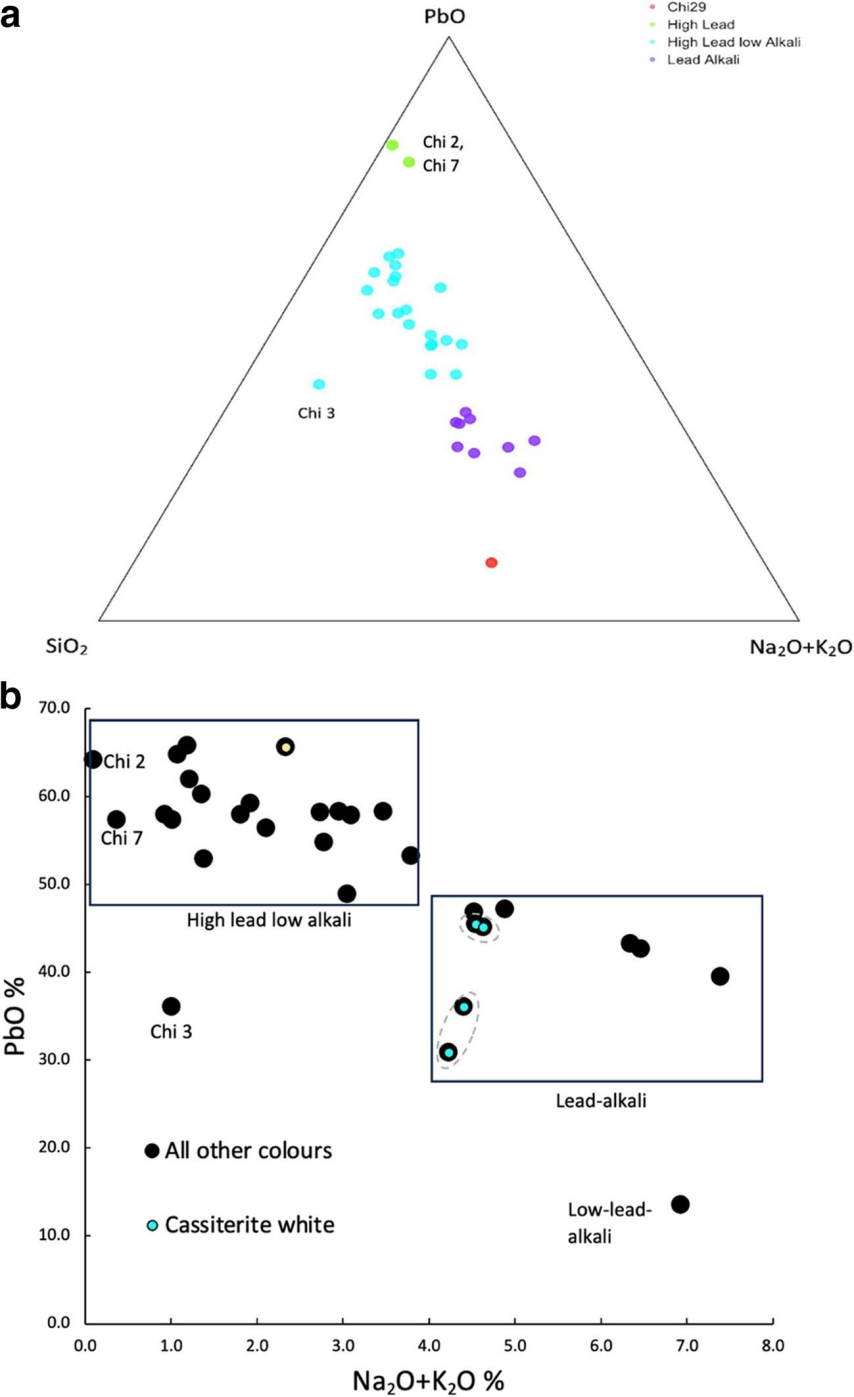
Plots showing the composition of the glazes analysed. **a**) Ternary diagram showing the major chemical compositions for the high lead low alkali, lead alkali and one outlier. Samples Chi 2, 7 and 3 present a weathered glaze. Chi 27 is opaque yellow. **b**) Biplot of 15b Owt% vs Na_2_O + K_2_O wt% for the glazes analysed showing that the cassiterite opacified samples are enriched in alkalis. Two areas (and thus two points) of samples Chi 6 and Chi 30 are shown in graph b, to take into account the inhomogeneity due to the presence of tin-rich areas (encircled in graph b)

Chemical analysis of the glazes indicates that there are two main types: a high-lead low-alkali type, with PbO > 50 wt% and variable alkali contents which do not exceed 4% and a lead-alkali type with lower contents of lead oxide between 30 and 50% as well as higher levels of alkalis between 4 and 7% (Tab. 2; Fig. 5a). One sample (Chi 29) is of a low-lead low-alkali composition and might have been imported to Tunisia. Indeed, even though its fabric shows textural features comparable to our Fabric 3, its Na_2_O and MgO contents are rather high, while its Fe_2_O_3_ content is lower than that of the other samples of this petrographic group (Tab. 2; Fig. 5a). In the absence of comparable published fabrics for this sample, it is not possible to infer about its provenance at this stage. Two samples (Chi 2 and 7) show a very low content of alkalis (< 1%), combined with elevated concentrations of P_2_O_5_ (> 9%) and CaO (> 7%), while PbO is still present in amounts well above 50% (Tab. 2; Fig. 5a). Under the SEM, these two samples appear severely weathered. A depletion in alkalis and a strong enrichment in P_2_O_5_, CaO, PbO is commonly observed in weathered lead glazes and glasses due to the leaching of alkali, the precipitation of calcium phosphate and the formation of lead and phosphorus compounds (Freestone et al. 1985). Three samples (Chi 6, Chi 30, Chi 27) contain higher levels of tin, and show evidence of particles of tin oxide and other tin compounds in the SEM.

All three cassiterite-opacified samples (discussed in detail below) present higher levels of alkalis (Na_2_O + K_2_O) relative to most other samples and are of the lead-alkali type (Fig. 5b). This might be explained by the addition of alkalis to obtain a white opaque glaze. Experiments on the production of lead–tin-oxide and tin oxide opacifiers in glazes have shown that the mixing of a Pb_2_SnO_4_ containing calx (Pb/Sn > 3) with silica and alkalis and its firing to a temperature above 750° C resulted in the dissolution of the lead–tin oxide and consequent precipitation of tin oxide crystals, resulting in a white opaque alkali frit (Matin 2019; Matin et al. 2018). This appears to have been the main method of production of white opaque glazes in eighth century Egypt and the Levant and continued to be in use until the nineteenth century in other regions in the Middle East, Europe and Central Asia (Matin et al 2018).

**Table 2:** Normalised chemical composition of the glazes determined by SEM–EDS

Sample	Ceramic Type	Glaze colour	Transparent/Opaque	Glaze Type	Na_2_O	MgO	Al_2_O3	SiO_2_	P_2_O_5_	Cl	K_2_O	CaO	TiO_2_	MnO	Fe_2_O_3_	CuO	ZnO	SnO_2_	Sb_2_O3	PbO	Na_2_O + K_2_0	Other
Chi 1	4c	Light green-turquoise and brown	T	High lead low alkali	0.8	0.4	2.2	31.8	0.0	0.0	2.3	3.1	0.2	0.0	0.7	0.5	0.0	0.0	0.0	57.9	3.1	
Chi 2 (weathered)	5	Light green-turquoise	T	High lead low alkali	0.0	0.1	1.1	13.7	9.7	0.0	0.1	7.6	0.2	0.0	1.0	0.8	0.0	0.0	0.0	64.2	0.1	
Chi 3 (weathered)	3a	Light green-yellowish	T	High lead low alkali	0.1	0.3	4.5	43.1	0.8	0.0	0.9	2.5	0.3	0.0	8.5	2.2	0.0	0.8	0.0	36.1	1.0	
Chi 4	3b	Light green-turquoise	T	High lead low alkali	1.2	0.2	0.6	33.5	0.0	0.0	1.5	2.5	0.0	0.0	0.7	1.2	0.0	0.3	0.0	58.2	2.7	
Chi 7 (weathered)	3a	Light green-turquoise	T	High lead low alkali	0.0	0.2	2.5	12.0	12.7	0.0	0.3	10.8	0.2	0.0	2.6	0.8	0.0	0.2	0.0	57.3	0.4	
Chi 9	1b	Yellow, green and brown	T	High lead low alkali	1.1	0.4	2.8	33.8	0.0	0.0	1.7	2.3	0.3	0.0	1.3	1.5	0.0	0.0	0.0	54.8	2.8	
Chi 10		Light brown, ochre, and dark brown	T	High lead low alkali	0.3	0.2	0.5	28.4	0.0	0.0	0.8	2.1	0.1	0.2	0.4	2.3	0.0	0.0	0.0	64.8	1.1	
Chi 11		Yellow and green	T	High lead low alkali	1.4	0.4	2.4	35.3	0.0	0.0	2.4	2.2	0.2	0.0	1.5	1.0	0.0	0.0	0.0	53.3	3.8	
Chi 14	4b	Yellow	T	High lead low alkali	1.4	0.3	3.8	30.2	0.0	0.0	2.1	2.0	0.3	0.0	1.3	0.3	0.0	0.0	0.0	58.3	3.5	
Chi 25		Yellow and dark green	T	High lead low alkali	0.2	0.3	3.0	30.0	0.0	0.0	0.7	1.4	0.2	0.0	1.4	4.5	0.0	0.1	0.0	58.0	0.9	0.3 ZnO
Chi 23	Ring base	Yellow, green and brown	T	High lead low alkali	0.3	0.2	2.6	33.8	0.0	0.0	0.8	0.5	0.1	0.0	1.0	3.4	0.0	0.0	0.0	57.3	1.0	
Chi 13	1b	Yellow, green and brown	T	High lead low alkali	0.6	0.1	0.2	27.0	0.0	0.0	0.6	0.6	0.0	0.0	0.1	4.9	0.0	0.0	0.0	65.8	1.2	
Chi 15	1b?	Yellow, green and brown	T	High lead low alkali	1.4	0.4	3.8	35.4	0.0	0.0	1.6	3.3	0.5	0.0	2.9	0.0	0.0	0.0	0.0	50.7	2.9	
Chi 17	Flat base	Yellow, green and brown	T	High lead low alkali	0.4	0.5	5.0	34.0	0.0	0.0	1.0	2.3	0.5	1.7	1.6	0.0	0.0	0.0	0.0	52.9	1.4	
Chi 18		Yellow, green and brown	T	High lead low alkali	0.5	0.2	1.7	33.7	0.0	0.0	1.6	1.5	0.1	1.4	1.2	1.4	0.3	0.0	0.0	56.4	2.1	0.4 ZnO
Chi 19	1b	Yellow and green	T	High lead low alkali	0.4	0.3	2.5	27.7	0.0	0.0	0.8	3.5	0.2	0.0	1.2	1.4	0.0	0.0	0.0	61.9	1.2	
Chi 20	1b	Green	T	High lead low alkali	1.0	0.5	3.6	36.5	0.0	0.0	2.1	2.8	0.3	0.0	1.4	3.0	0.0	0.0	0.0	48.9	3.0	
Chi 21	1b	Yellow and green	T	High lead low alkali	0.4	0.3	2.9	29.9	0.0	0.0	1.0	1.7	0.3	0.0	1.4	1.8	0.1	0.1	0.0	60.3	1.4	0.5 ZnO
Chi 22	2	Yellow, green and brown	T	High lead low alkali	0.4	0.2	1.4	32.8	0.0	0.0	1.5	2.0	0.0	1.7	0.6	0.0	0.0	0.0	0.0	59.2	1.9	
Chi 26		Blue?, brown	T	High lead low alkali	0.4	0.4	2.4	34.0	0.0	0.0	1.4	1.2	0.2	0.0	1.3	0.7	0.1	0.0	0.0	57.9	1.8	
Chi 27	Ring base	Light yellow, green and brown	O	High lead low alkali	1.0	0.1	0.4	26.1	0.0	0.0	1.3	1.2	0.0	0.0	0.3	1.4	0.1	2.5	0.0	65.6	2.3	0.4 ZnO
Chi 28	3a	White, green and brown	O	High lead low alkali	0.4	0.2	0.9	28.9	0.0	0.0	1.0	1.6	0.0	2.4	0.4	3.8	0.0	0.0	0.0	60.5	1.3	
Chi 30 Sn rich	6	White and brown	O	Lead alkali	0.9	0.2	1.8	34.5	0.0	0.0	3.3	1.6	0.0	3.2	0.7	0.3	0.0	22.5	0.0	30.9	4.2	
Chi 30	6	White and brown	O	Lead alkali	0.8	0.4	2.6	41.1	0.0	0.0	3.6	3.7	0.1	8.0	1.0	0.1	0.0	2.5	0.0	36.1	4.4	
Chi 5		Light green-turquoise	T	Lead alkali	1.1	0.3	2.0	39.0	0.0	0.0	3.4	3.4	0.2	0.0	1.4	1.5	0.5	0.3	0.0	46.8	4.5	0.5 ZnO
Chi 6	5	Light green-turquoise	O	Lead alkali	0.8	0.1	2.1	42.8	0.0	0.0	3.7	2.6	0.0	0.0	0.6	1.8	0.0	0.0	0.0	45.4	4.5	0.3 ZnO
Chi 6 Sn rich	5	Light green-turquoise	O	Lead alkali	0.7	0.1	1.9	42.3	0.0	0.0	3.9	1.7	0.1	0.0	0.5	1.9	0.1	1.8	0.0	45.1	4.6	
Chi 16		Yellow	T	Lead alkali	2.0	0.3	3.1	40.6	0.0	0.0	2.9	1.9	0.3	0.0	1.7	0.0	0.0	0.0	0.0	47.2	4.9	
Chi 12	4a?	Yellow and brown	T	Lead alkali	2.8	0.6	5.0	38.8	0.0	0.0	3.6	2.4	0.3	0.4	2.9	0.0	0.0	0.0	0.0	43.2	6.3	
Chi 8	4a	Yellow	T	Lead alkali	4.1	1.4	8.7	30.9	0.0	0.0	2.4	5.6	0.5	0.0	3.7	0.0	0.0	0.0	0.0	42.6	6.5	
Chi 24	Ring base	Light yellow	T	Lead alkali	2.4	0.5	3.5	42.3	0.0	0.0	5.0	3.2	0.3	0.0	2.7	0.2	0.0	0.5	0.0	39.5	7.39	
Chi 29	3a	White?	T	Alkali low lead	1.3	2.4	12.6	52.8	0.0	0.0	5.6	6.8	0.5	0.0	3.8	0.7	0.0	0.0	0.0	13.5	6.9	

#### Transparent glazes

The ceramic wares decorated with transparent glazes can be classified into monochrome and polychrome. Monochrome wares are yellow-amber or light green in colour, while polychrome ones have dark brown and/or green decorations with a yellow, light green or turquoise background. All the glazes analysed, except for three samples (Chi 16, Chi 12 and Chi 8), are of the high lead low alkali types (Fig. 5b). The glaze of sample Chi 3 is heavily weathered and presents significant loss of PbO (Tab. 2). The yellow glazes have been obtained by adding iron oxide (1–3% Fe_2_O_3_), while the green glazes have been coloured with copper (1–3% CuO) completely dissolved in the glaze. The brown glazes and brown decorations have been made using manganese in variable quantities (0.4–8%) (Tab.2). A light green glaze was due to the combination of few amounts of Fe_2_O_3_ and CuO (around 1–1.5%), while turquoise is obtained adding copper to a glaze containing some alkalis (Tab. 2). Zinc oxide has also been identified in some of the samples coloured with copper (Chi 1, Chi 5, Chi 6, Chi 18, Chi 25, Chi 27) which might point to the use of brass as source of copper for these samples, but it might also have been introduced as an impurity with the lead source.

**Fig. 6:**
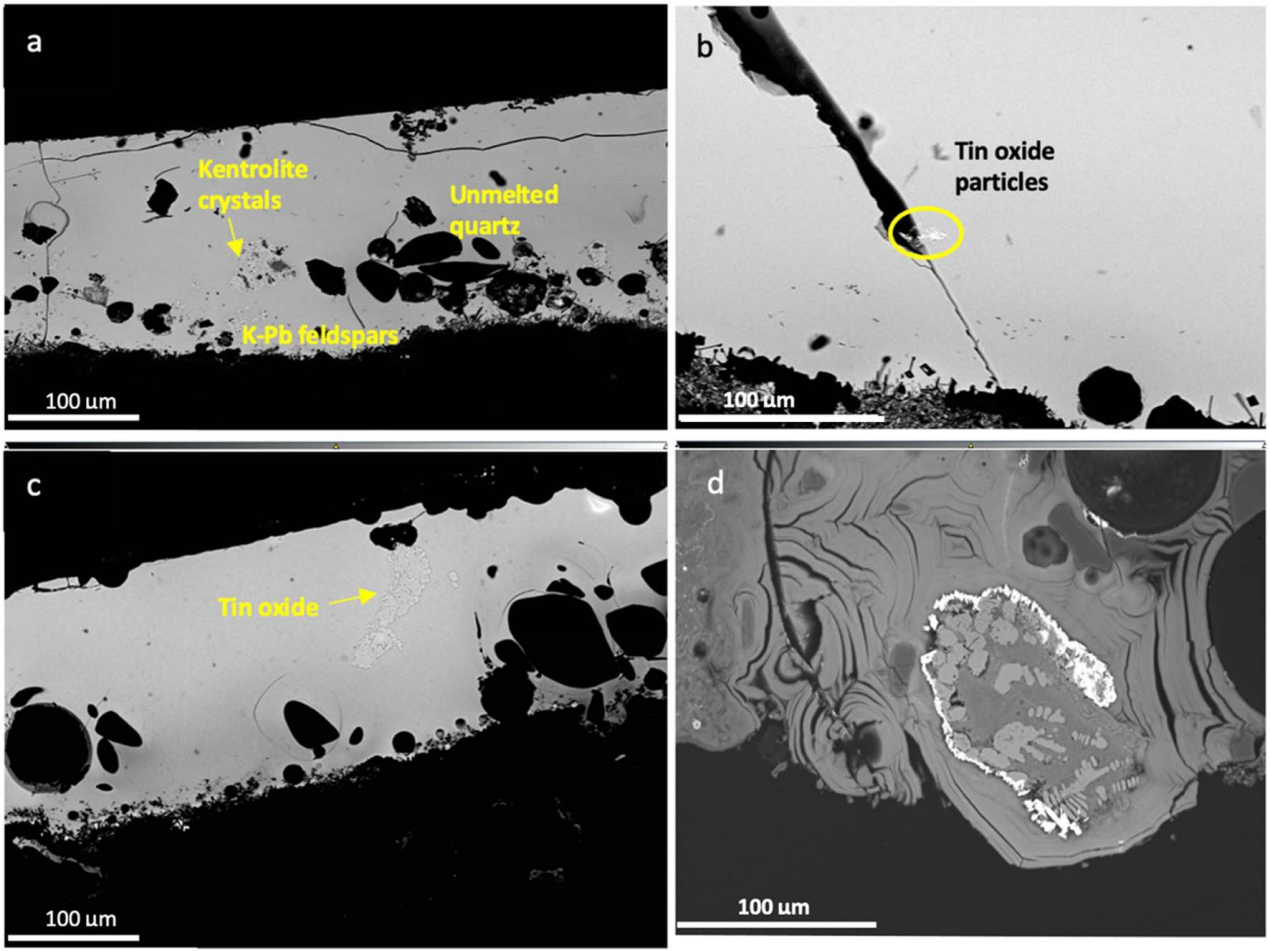
BSE micrographs of the polychrome transparent glazes analysed showing: **a**) detail of a cross section of a yellow and brown glaze showing kentrolite particles (marked with an arrow), unmelted quartz and K-Pb feldspars at the ceramic interface; **b**) the rare occurrence of tin oxide (or tin and antimony) particles in the transparent glazes; **c**) the presence of a large agglomerate of tin oxide particles in sample Chi 26; **d**) an iron slag inclusion in sample Chi 3

The microstructure of the glazes was investigated under the SEM. Glaze layers vary between 70 and 200 μm in thickness and are characterised by the presence of frequent residual quartz grains and few bubbles (Fig. 6a,c). At the glaze-ceramic intersection it is possible to observe the presence of dark crystallites in many glazes: these are crystals of lead-potassium feldspars which form due to the interaction between the glaze and the ceramic body (Molera et al. 2001; Walton 2004), as confirmed by the SEM–EDS analysis of the crystals (Fig. 6a). In the brown decorated areas, it is also possible to see the presence of kentrolite (Fig. 6a), a lead manganese silicate (Pb_2_Mn_2_Si_2_O_9_), which forms commonly in lead glazes (Molera et al. 2022; Pradell and Molera 2020; Walton 2004). Kentrolite is formed due to the reaction of manganese oxide with lead and silica oxides in the surrounding glaze. As kentrolite melts at temperatures above 900°, its presence in the glaze usually indicates a firing temperature below 900 °C (Molera et al. 2022, 2013).

Interestingly, virtually all the transparent glazes reveal the presence of very rare and small particles rich in SnO_2_, PbO, and sometimes Sb_2_O_5_, which often results in low concentrations (< 0.8%) of these oxides in the glaze (Fig. 6b). Given the rare occurrence and small size of the crystals, their intentional addition as opacifiers can be excluded. Most likely, these are newly formed crystals that have been introduced as impurity with the lead used to make the glaze, as will be discussed below. In contrast to these findings, one sample (Chi 26) shows one large agglomerate (ca 100 μm) of SnO_2_ particles (Fig. 6c) The glaze of this sample has been coloured with iron and copper oxides and has a light blue appearance, but it is not very well preserved. It is difficult to determine whether the presence of SnO_2_ in this sample is related to a failed attempt to obtain an opaque white background, or whether this is also due to its introduction as an impurity of the lead flux used.

Particularly remarkable is the light green sample Chi 3, which has an exceptionally high iron content, of an inclusion showing large irregular grains of wüstite (FeO) dendrites (Fig. 6d). Spot analysis of the dendrites shows a composition typical of wüstite. This is likely to be a slag derived from smelting iron ores (or smithing). Particles of metallic lead, iron and silicon oxide can be seen surrounding this inclusion, likely formed due to the reaction of the iron slag with the lead glaze.

#### Opaque glazes

**Fig. 7:**
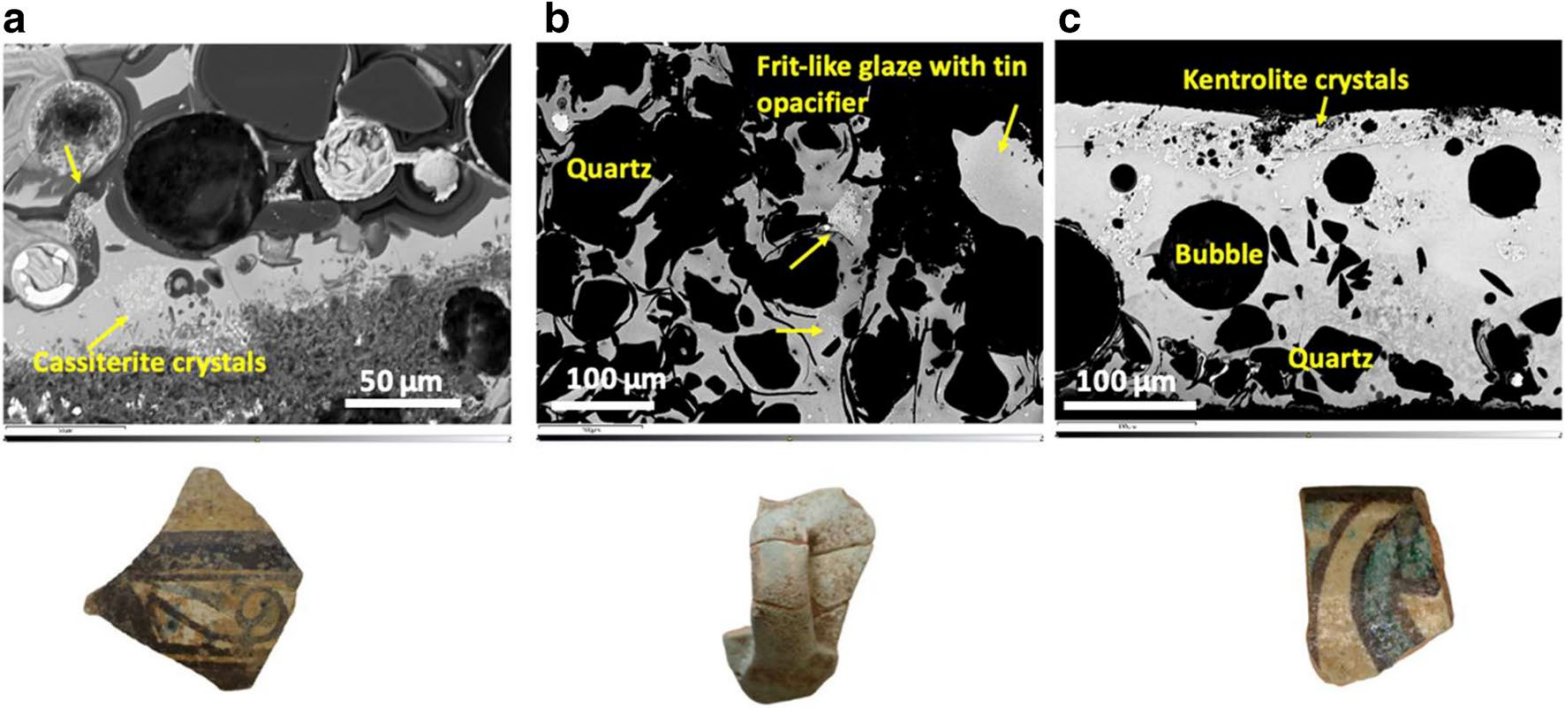
BSE micrographs of the opaque glazes analysed with picture of the respective sherd showing: **a**) the abundant presence of cassiterite crystals as opacifiers (Chi 30); **b**) the use of tin opacifier in a frit-like glaze showing numerous unmelted quartz grains in the glaze (Chi 6); **c**) the presence of large bubbles, unmelted quartz and overglaze brown decoration with associated kentrolite crystal near the surface of the glaze in sample Chi 28. This sample was likely opacified using quartz rather than tin oxide

All the wares showing a macroscopically opaque glaze (Chi 6, Chi 27, Chi 28, Chi 30) are polychrome, except for sample Chi 6 which is of a turquoise colour with no apparent additional decorations. The opaque polychrome glazes analysed are turquoise, white and one yellow glaze, and they present dark brown and/or green decorations. The turquoise (Chi 6) and the one white glaze (Chi 30) are of the leadalkali type and were opacified with cassiterite crystals which are clearly visible in the SEM micrographs (Fig. 7a, b). In contrast to the transparent glazes, in these samples the cassiterite crystals are more numerous and of a bigger size, pointing to their intentional addition as opacifier. Bulk SEM–EDS measurements confirm the presence of tin oxide as opacifier (SnO_2_ **≈** 1–2.5%) (Tab. 2). Nevertheless, the tin oxide particles tend to be heterogeneously distributed in the glazes, with aggregates of particles in the areas where these were originally present, and this results in a relatively wide range of SnO_2_ contents even within the same glaze depending on the area analysed. As in the transparent glazes, the brown areas are due to the addition of MnO and show the presence of kentrolite particles, while the turquoise glaze is coloured by copper oxide. Sample Chi 6 is a unique case: the presence of numerous large unreacted quartz grains (ranging from 50 to 250 μm in size) in the glaze suggests that this was a frit consisting of a mixture of lead oxide, quartz sand, tin oxide, copper oxide and alkalis which was not fully molten. Tin is incorporated in the frit in the form of cassiterite to enhance the opacity of the glaze (Fig. 7c). In addition to tin oxide, a white opaque effect was also achieved adding quartz particles (Chi 28), a method also documented in Bir Ftouha and Utica (Salinas et al. 2022b, 2020).

**Fig. 8:**
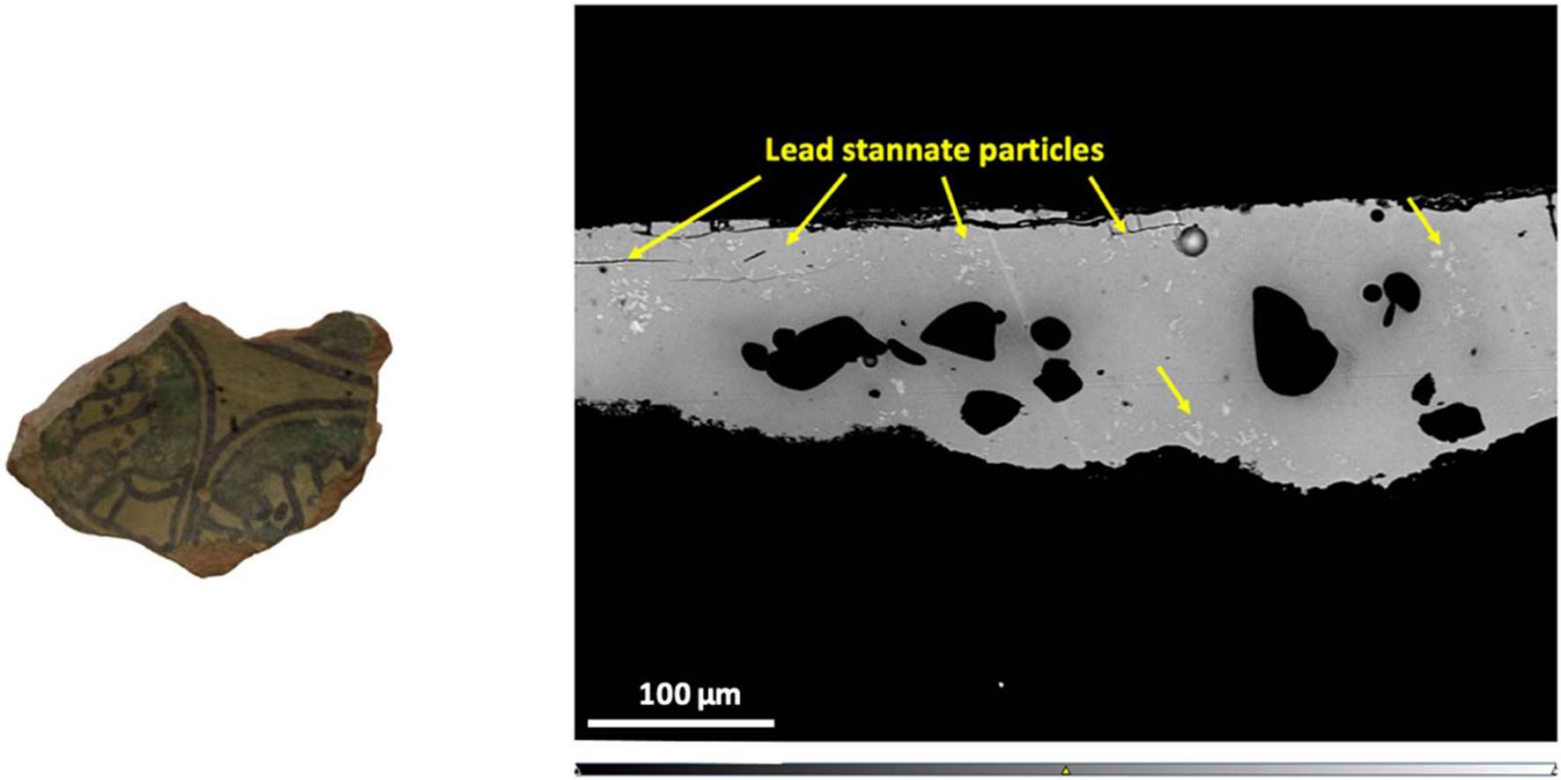
BSE image showing lead stannate particles in sample Chi 27 (right) and picture of the sample analysed showing the surface decoration and the light-yellow background (left)

One sample (Chi 27), a petrographic outlier presenting a light-yellow glaze which looks opaque macroscopically, is of particular interest. In contrast to the other opaque glazes, it is of the highleadlow alkali type, with a negligible amount of alkalis of 2.5% (Tab. 2). The yellow glaze contains 2.5% tin oxide (Tab. 2), and lead stannate particles (Pb(Sn,Si)O_3_) can be clearly seen in the yellow glaze area which are responsible for its opaque yellow colour (Fig. 8). The glaze has a Pb/Sn ratio of 32, representing the lead and tin calx composition. Furthermore, iron oxide is present in very low amounts of 0.3% in this glaze compared to the other yellow samples (Tab. 2), suggesting that only lead stannate was added intentionally as a colourant. This is the first published example of lead-stannate opacified glaze in Tunisia. Importantly, a recent study by Matin et al. (2018) on Islamic glazes from the Levant, Egypt and Mesopotamia, has demonstrated that yellow glazes differ from white glazes in having higher contents of PbO (> 60%), negligible alkali (usually < 2% Na_2_O + K_2_O), and a high Pb/Sn ratio between 16 and 54, thus being in good agreement with our results. The importance of having a high lead glaze composition for the stability of the lead stannate particles was also previously demonstrated by Tite et al. (2008).

#### Glazing method

**Fig. 9:**
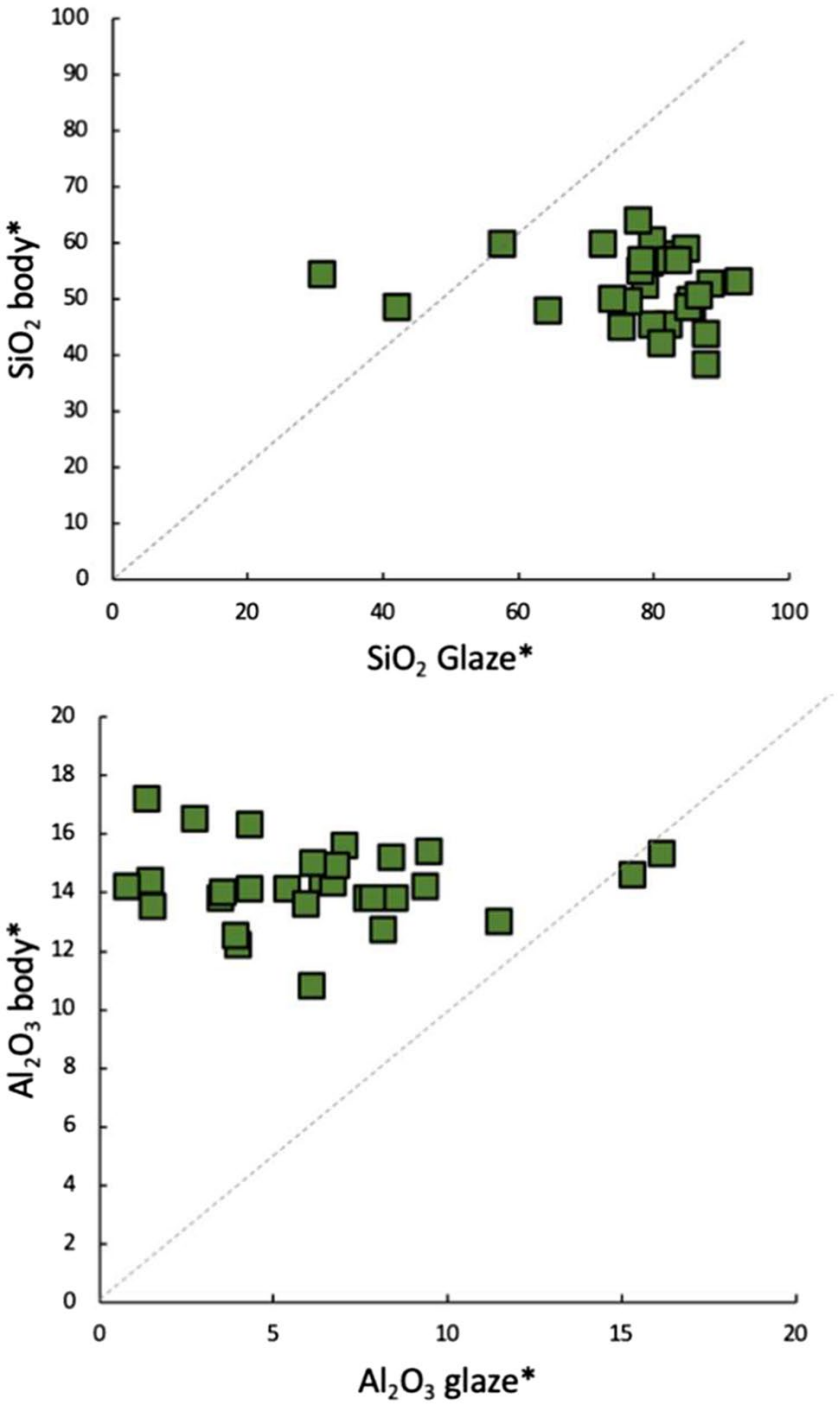
Body-glaze comparison plots for SiO_2_ and Al_2_O_3_ contents after subtraction of PbO and normalising the composition

The two main methods to apply a lead glaze are either to apply a lead compound directly to the surface of the ceramic, or to apply a mixture of lead and silica. In both methods, the glaze could be applied to a biscuit fired or to an unfired ceramic body (Tite et al. 1998). As demonstrated by Hurst and Freestone (1996), a simple way to assess whether a lead compound by itself or a lead-silica mixture was used is to subtract the lead content from the glaze composition and then to recast to 100%. If the resulting composition matches that of the body, PbO alone has reacted with the ceramic body, while if a lead-silica mixture was used, the resulting composition will depend on the relative diffusion rates of the body components into the glaze. In the great majority of the samples analysed, the glaze was obtained using a lead-silica mixture, as supported by the observation that most of the samples do not fall on the unity slope line on the biplots in Fig. 9, which compare the SiO_2_ and Al_2_O_3_ contents of the glazes and their respective bodies after subtraction of PbO and renormalisation of the data. Three samples (sample Chi 8, Chi 2 and Chi 7) which fall on or near the unity slope, presented a substantial alteration of the glaze, with the layered structure typical of weathered glazes, making their composition unreliable.

The majority of samples (27/30) present a thin interface between the glaze and the body (< 30 μm), compatible with a double firing where the glaze mixture was applied to a biscuit-fired body (Molera 1996; Molera et al. 2001). Only three samples (13, 17 and 23) present a thicker interface (between 30 μm and 100 μm), richer in potassium-lead-aluminium silicate crystals, which might be indicative of a single firing (Molera 1996; Molera et al. 2001; Tite et al. 1998). It should be noted that different factors, such as the temperature at which the glaze was fired and the lead content of the glaze, may have an effect on the interface microstructure (Molera et al. 2001; Walton 2004).

## Discussion

**Fig. 10:**
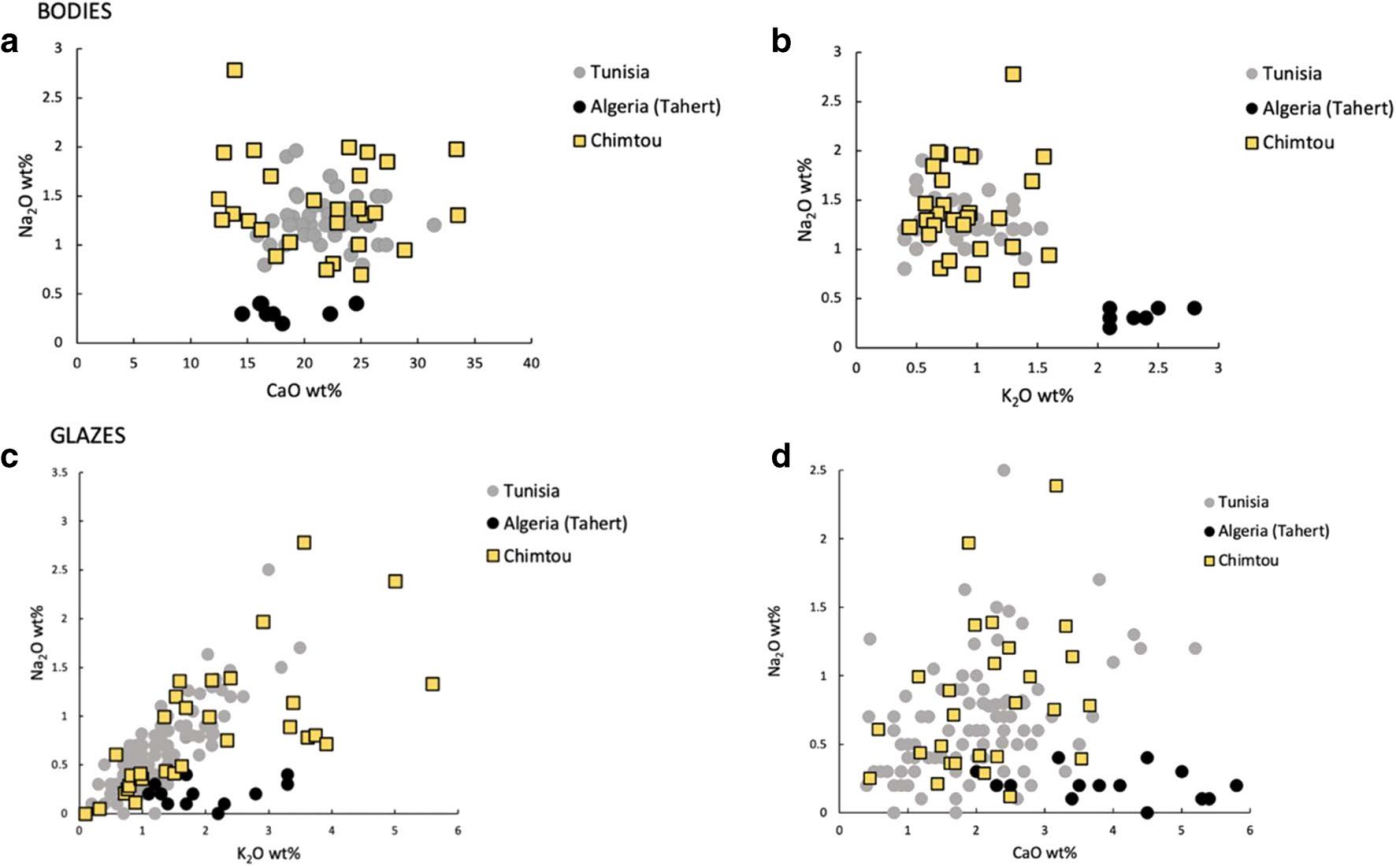
Comparison between Na_2_0 vs CaO and Na_2_O vs K_2_O contents of the bodies (**a**, **b**) and glazes (**c**, **d**) from Tunisia (data from: Ben Amara et al. 2001; Ben Amara et al. 2005; Salinas et al. 2022b, 2020), Algeria (data from: Djellid et al. 2023) and Chimtou (analysed in this paper)

The mineralogical and textural characteristics of the samples suggest a central or north-eastern Tunisian origin for the three main fabric groups identified. The predominant presence of quartz, calcareous inclusions and microfossils in all fabrics is consistent with the use of early cretaceous calcareous fossiliferous clays from north-eastern and central Tunisia (the latter is the Kairouan-Raqqada-Sabra production) (Capelli et al. 2011), however, these sediments are widespread in Tunisia, and it is very difficult to securely identify the production location without the sampling and analysis of potential clay sources (Capelli et al. 2011; Capelli and Bonifay 2014). Indeed, fabrics of comparable composition and texture to our Fabrics 1, 2 and 3 are found at Raqqada and Sabra al Mansuriyya (Ben Amara et al. 2001; Ben Amara et al. 2005; Group 1 in Capelli et al. 2011). Similar fabrics, which vary from cream/greenish to red in colour, are also found at Utica and Bir Ftouha and linked to the Kairouan production, though a coastal origin is not excluded (Salinas et al. 2022b, 2020). Testolini (2018:130–135) also reported similar fabrics in her study of early medieval Sicilian ceramic assemblages which she interpreted as imports from North Africa, likely made in different (unidentified) workshops. In terms of their chemical composition, our samples plot with the Tunisian ceramic bodies (Fig. 10b), showing very similar CaO and Na_2_O concentrations which further suggest a Tunisian provenance. (Fig. 10a). They differ from the Algerian samples by having higher Na_2_O and lower K_2_O (Djellid et al. 2023), perhaps highlighting a different technology (i.e. the use of sea water in forming the paste).

All samples, except one, have a lead-rich glaze with a variable amount of alkalis. The majority (26/30) of samples have a transparent high-lead glaze, with an average content of 57.3 wt% PbO, 30.5 wt% SiO_2_, 2.31 wt% Al_2_O_3_, and alkali levels (Na_2_O + K_2_O) < 4% (Glaze Type 1). Iron, copper and manganese colorants were used as colourants for the transparent glazes, typical of early Islamic glaze production (Ben Amara et al. 2001; Pradell and Molera 2020; Tite 2011). These data are consistent with our current understanding of the production of medieval high-lead glazes in the eastern Mediterranean, and parallel results from roughly contemporary Tunisian assemblages at Utica, Bir Ftouha, Sabra al-Mansuriya and Raqqada (Ben Amara et al. 2001; Ben Amara et al. 2005; Salinas et al. 2022b, 2020). The closest compositional match for the transparent glazes are the polychrome transparent glazes from Sabra al Mansuriya (Capelli et al 2011), which are also characterised by a more variable alkali content which does not exceed 4 wt% and by similar levels of PbO between 50% and 60 wt%. The Chimtou transparent glazes tend to have slightly higher alkali contents than those from Utica and Bir Ftoua (typically < 2%) and thus are more similar to those from Sabra and Raqqada, even though the latter present lower PbO contents which are generally < 52 wt %.

A smaller group of seven samples, which encompasses all samples of petrographic Fabric 2- including the glazes opacified with cassiterite- two outliers and sample Chi 16, have lower contents of lead oxide (PbO < 50%) and higher contents of alkali which reach 7.39 wt%. This group is comparable to a group of lead-alkali glazes opacified with cassiterite crystals from Sabra al-Mansuriya and believed to be a central Tunisian production (Capelli et al 2011). Therefore, our results suggest that both groups of glazes were likely produced in Tunisia.

While the presence of a thin glaze-body interface in most of the samples suggests that the glaze mixture was applied to a biscuit-fired body, a small number of samples have a thicker interface and can be associated to a single firing. Both production processes are also documented by Salinas et al. (2020) at Bir Ftouha. The glazes analysed also show a positive correlation between Na_2_O and K_2_O, similarly to other published Tunisian glazes (Fig. 10c). This correlation was first observed by Djellid et al. (2023), who suggested that it might reflect the use of salty water to bleach the ceramics, as the brines found in many Tunisian salt lakes are enriched in sodium and potassium. Tunisian glazes also have higher Na_2_O and lower CaO than the western Algerian ones (ibid), a tendency that can be observed in our samples (Fig. 10d).

Several interesting observations can be made. First, tin-based opacifiers have been identified in virtually all opaque glazes analysed. The question of when and how tin reached Tunisia is still highly debated. Our samples are dated broadly to 9th to the 12th centuries and therefore it is impossible to test the hypothesis of Salinas and colleagues (2019a, 2020) that tin opacification was introduced to Tunisia after the Fatimid conquest of Egypt in 969. However, it is important to note that in Sicily, a region whose glaze technological developments are traditionally thought to follow Tunisia, the use of tin oxide as white opacifier in glazes is documented in the first half of the tenth century (in early Fatimid contexts) (Capelli et al. 2020).

As discussed above, sample Chi 27 was coloured and opacified using lead-stannate and thus resembles yellow glaze technology initially used in Egypt and Syria in the late seventh and eighth centuries and subsequently in Iraq in the ninth century and in Iran and Central Asia in the ninth-tenth century and beyond (Matin et al 2018). In Egypt there was a shift to the use of lead-antimonate in the ninth-eleventh centuries, which appears to have been also adopted in Tunisia in the eleventh century (Salinas et al. 2022b). In this context, the discovery of a glazed ceramic in Chimtou coloured and opacified using lead-stannate raises questions about whether this glaze was manufactured locally, or if this particular vessel was imported. From a purely stylistic perspective, the glaze has the traditional geometric decorative patterns of Tunisian ceramics. The glaze compositional make-up is somewhat similar to that of published Central Asian examples, such as ninth-tenth century glazes from Nishapur and Merv (Matin 2016), however, the body compositions are different. Nishapur ceramics are characterised by sediments associated with low igneous and metamorphic rocks, while the sherds from Merv are fine-grained with a silty-alluvial fabric (Matin 2016). In contrast, sample Chi 27, a petrographic outlier, was manufactured using a calcareous clay, rich in quartz associated with large inclusions of micritic limestone fragments as well as microfossils. An exact match for this type of paste has not been found, however a certain similarity with fabrics from the Dougga region (e.g. Bonifay et al. 2002–2003) could suggest an origin in the wider Medjerda Valley. The geological features of the Valley characterised by the presence of Mesozoic and Cenozoic sedimentary series are consistent with its inferred north-western Tunisian provenance (Capelli and Bonifay 2014). Thus, if a Tunisian manufacture for this glaze is correct, the use of lead stannate might suggest that this technology was continuously used in Tunisia since it was first employed in Egypt and the Levant in the seventh-eighth centuries. Certainly, more analyses targeting Tunisian opaque yellow glazes are needed to answer these questions, but these initial results suggest we need to be cautious about assuming a late Egyptian-Fatimid origin of tin-based opacification technology in North Africa.

Minor amounts of tin oxide and tin-antimony particles were identified in the majority of transparent lead glazes. The size and rare occurrence of the particles suggest that their addition as an opacifier is unlikely. Importantly, Sn amount in galena ores (PbS), one of the traditional lead ores employed as a source of lead for glazing (Pradell and Molera 2020; Salinas et al. 2019b), can differ depending on the mining area, but usually is < 100 mg kg^−1^ (Gomes et al. 2016a). Thus, we propose that tin oxide and tin-antimony compounds were introduced using scrap metal as a source of lead, such as Roman lead pipes (fistulae acquariae) which were joined with a Pb–Sn solder and contained antimony as an impurity (Gomes et al. 2016a,b; Wyttenbach and Schubiger 1973). Lead was used widely in the Roman and medieval world, mainly for industrial purposes, for example for aqueducts, pipes and water tanks, in toiletries, for tablets, for covering roofs, for shipping and fishing, in soldering and for handles (Boulakia 1972; Carroll et al. 2021; Müller et al. 2015),—and all these could have been collected as scrap metals. Assuming that the potters were glazing their pots in Tunisia mainly using scrap metal, how much scrap metal did they need and was this readily available? We have calculated that to cover a pot of 25 cm of diameter, with a glaze of 0.2 mm thickness, the potters would have needed approximately 130 g of lead metal (for 60% Pb by weight). To give an example, the weight of lead pipes ranged from ca. 27 kg (*fistula quinarian*) to ca. 435 kg (*fistula centenaria*) (Boulakia 1972), which would provide enough lead for between 207 to 3000 pots. The use of scrap metal as a source of lead has also been suggested for the production of glazed ceramics from Middle Islamic Jerash (Ting et al. 2019), suggesting that this was a solution embraced by potters in different regions of the Islamic world.

A decline in the availability of raw materials might result in an increased reliance on recycled materials (Barfod et al. 2018; Duckworth and Wilson 2020; Freestone 2015; Keller 2005; Paynter and Jackson 2016). In North Africa, however, lead was not a scarce material: it was produced from galena as a leftover from silver mining, and it was mined separately (Skaggs et al. 2012; Tissot 1884; Berthon 1922; Wolf et al. 2003). Documentary, literary and archaeological sources provide abundant evidence for the exploitation of lead mines in Tunisia during antiquity and the middle ages (Ben Hadj Naceur Loum 2020:253; Tekki 2020). Lead deposits for silver mining were exploited in North Africa between the second half of the fourth and the beginning of the third century BC (Berthon 1922; Delile et al. 2019; Fenn et al. 2009; Skaggs et al. 2012; Tissot 1884). Lead isotopic data on Abbasid silver dirhams has identified a still unrecognised ore source, with lead isotopes ratios consistent with lead-based deposits in Tunisia (the Nappe zone in northern Tunisia), although there is an overlap with little known deposits in Morocco (Merkel et al. 2023). While it is likely that future research will provide more conclusive evidence of medieval lead-mining in Tunisia/ eastern Algeria, the many lead objects in late antique towns were certainly an easily accessible and economically convenient sources of lead. This does not imply that craftsmen did not have access to other sources of lead – lead was widely traded in both the Roman and Islamic world (Bode et al. 2021; Boulakia 1972; Carroll et al. 2021; Klesner et al. 2021; Trincherini et al. 2009; Wolf et al. 2003) – but rather it testifies a continuation of a practice of reuse and recycling of obsolete objects which was a normal part of economic and social life.

Evidence of recycling can be traced in medieval ceramic, glass and metal (Barfod et al. 2018; Bottaini et al. 2022; Milwright 2021) and broken or damaged metals were widely collected as scrap metal and melted down, as also confirmed chemically due to the introduction, dilution or loss of components compared to an unrecycled object (Bottaini et al. 2022; Gener et al. 2014; Orfanou et al. 2020; Wulff 1966). The collection and stockpiling of scrap metal is documented by archaeological evidence from the Levant, such as a hoard in Fatimid Tiberias containing around 200 kg of scrap metal which was most likely stored for recycling (Ponting 2008) and a small collection of broken metal objects in a wooden box in Umayyad Jerash (Lichtenberger et al. 2017). In Jerusalem, a salvage excavation of the Givati Parking Lot uncovered an Umayyad metallurgical workshop with numerous copper-alloy Byzantine objects, and it has been suggested these were used as scrap and recycled (Tchekhanovets 2018). Likewise, the Cairo Geniza documents provide evidence that “old or broken vessels and implements of all descriptions were sent from Aden to India and worked there into new utensils” (Goitein and Friedman 2008:16). The collection of discarded materials could even be a specialised activity, and written sources describe individuals who specialised in hunting on the streets and city dumps for materials to sell to craftsmen (see Milwright 2021).

Finally, one sample (Chi 3) showed the addition of an iron slag as probable source of iron to colour the glaze. Its use might point to a close connection between glaze making and metallurgy, similar to that observed in Early Umayyad Spain (Salinas et al. 2022a; Schibille et al. 2020) as well as providing evidence of an advanced empirical understanding of material properties and experimentation with metallurgical by-products. Interestingly, in his comprehensive study on Persian traditional crafts, Wulff (1966: 162) reports that Persian potters bought iron from the blacksmith in the form of hammerscale to colour the glaze yellow, pale green and green (mixed with copper). It is thus reasonable to assume that similar cross-craft interaction took place in medieval Tunisia as well as in other regions of the Islamic world.

## Conclusions

This analysis of a group of glazed ceramics from the medieval Chimtou provides new insights on the technological processes employed in their manufacture and Tunisian glaze production in the late ninth-twelfth centuries. Firstly, the Chimtou samples are all lead glazes with differing amounts of alkali, in line with contemporary Islamic glaze making technology. Secondly, all samples, which differ in terms of their texture, are characterised by a calcareous paste, rich in quartz inclusions and microfossils, strongly suggesting that these were made using calcareous ancient marine clay deposits, possibly from central or north-eastern Tunisia. Soda contents suggest that salt water was probably added during the formation of the paste to obtain a bleached surface. Three main petrographic fabrics have been identified based on different proportions of inclusions, which may indicate the existence of several regional workshops producing a relatively wide range of glazed ceramics in Tunisia. Two sub-fabrics differ only in terms of their colour, likely a result of diverse firing conditions. The chemical composition of both the ceramic pastes and glazes is comparable to other published Tunisian ceramics, notably those from the Kairouan region, further supporting a Tunisian provenance. Additionally, virtually all glazes reveal the presence of rare inclusions of Sn oxide and Sn-Sb compounds as impurities, which strongly suggests the use of scrap metal as a source of lead to manufacture the glaze. One sample shows the use of iron slag as a source of colourant for the glaze. Further work needs to consider the source and provenance of the metals employed in glaze making in relation to metallurgical production in North Africa, in order to understand interconnections between these two industries as well as the development of new innovations in glaze making and the possible transfer of technology. An important next step in the study of Tunisian glazed ceramics will be the LA-ICP-MS analysis of both the glazes and ceramic bodies from stratified assemblages at multiple sites in order to have a more detailed and accurate chemical characterisation of the raw materials used and to establish their provenance. Finally, though there is not conclusive evidence for an early use of a tin-based opacifier (before the late tenth century) in the samples analysed, one sample coloured and opacified with lead stannate and possibly manufactured in Tunisia, raises tantalising questions about the introduction of tin-opacification technology in North Africa.

### Supplementary information

Below is the link to the electronic supplementary material.

Supplementary file1 (DOCX 20 KB)

## Data Availability

Data is provided within the manuscript or supplementary information files.
