# Supplementary material for: The technology of polychrome glazed ceramics in Ifriqiya: new data from the site of Chimtou
Source: Archaeol Anthropol Sci. 2024 Mar 23;16(4):61. doi: 10.1007/s12520-024-01974-x (PMC10960904; doi:10.1007/s12520-024-01974-x)
Supplement: Supplementary file 1 — Supplementary file1 (DOCX 20 KB) [file 12520_2024_1974_MOESM1_ESM.docx]

**Table S1**: Chemical composition of glass reference materials as analysed and published. Expected values are mainly from Brill (1999) with additional information from Adlington (2017). The analysed result is the average of five analyses in wt% determined by SEM-EDS. Empty cells indicates ‘not published’ on the published data or below detection limit on the analysed data.

|  | **Na_2_O** | **MgO** | **Al_2_O3** | **SiO_2_** | **P_2_O_5_** | **SO_3_** | **K_2_O** | **CaO** | **TiO_2_** | **MnO** | **Fe_2_O_3_** | **CoO** | **CuO** | **ZnO** | **SnO_2_** | **Sb_2_O_3_** | **BaO** | **PbO** |
| --- | --- | --- | --- | --- | --- | --- | --- | --- | --- | --- | --- | --- | --- | --- | --- | --- | --- | --- |
| **Corning A** |  |  |  |  |  |  |  |  |  |  |  |  |  |  |  |  |  |  |
| Mean | 13.7 | 2.55 | 0.86 | 66.1 |  | 0.28 | 2.95 | 5.07 | 0.83 | 1.04 | 1.10 | 0.18 | 1.22 |  | 0.29 | 1.87 | 0.50 | 0.08 |
| sd | 0.28 | 0.07 | 0.03 | 1.44 |  | 0.04 | 0.07 | 0.12 | 0.04 | 0.05 | 0.04 | 0.03 | 0.05 |  | 0.09 | 0.10 | 0.06 | 0.06 |
| Expected | 14.3 | 2.66 | 1.00 | 66.6 | 0.08 | 0.14 | 2.87 | 5.03 | 0.79 | 1.00 | 1.09 | 0.17 | 1.17 | 0.04 | 0.19 | 1.75 | 0.46 | 0.07 |
| Absolute error | -0.56 | -0.11 | -0.14 | -0.45 |  | 0.14 | 0.08 | 0.04 | 0.04 | 0.04 | 0.01 | 0.01 | 0.05 |  | 0.10 | 0.12 | 0.04 | 0.01 |
| **Corning B** |  |  |  |  |  |  |  |  |  |  |  |  |  |  |  |  |  |  |
| Mean | 16.1 | 0.99 | 4.04 | 60.4 | 0.77 | 0.71 | 1.09 | 8.62 | 0.12 | 0.25 | 0.34 | 0.05 | 2.71 | 0.20 | 0.11 | 0.61 | 0.07 | 0.52 |
| sd | 0.35 | 0.04 | 0.09 | 1.08 | 0.05 | 0.04 | 0.03 | 0.15 | 0.03 | 0.03 | 0.03 | 0.03 | 0.07 | 0.05 | 0.07 | 0.08 | 0.04 | 0.05 |
| Expected | 17.0 | 1.03 | 4.36 | 61.6 | 0.82 | 0.49 | 1.00 | 8.56 | 0.09 | 0.25 | 0.34 | 0.05 | 2.66 | 0.19 | 0.02 | 0.46 | 0.08 | 0.61 |
| Absolute error | -0.90 | -0.04 | -0.32 | -1.11 | -0.05 | 0.22 | 0.09 | 0.06 | 0.03 | 0.00 | 0.00 | 0.01 | 0.05 | 0.01 | 0.09 | 0.15 | 0.00 | -0.09 |
| **Corning C** |  |  |  |  |  |  |  |  |  |  |  |  |  |  |  |  |  |  |
| Mean | 0.99 | 2.56 | 0.77 | 33.0 | 0.02 | -0.63 | 2.84 | 4.93 | 0.86 |  | 0.31 | 0.17 | 1.15 | 0.05 | 0.21 |  | 12.0 | 38.5 |
| sd | 0.05 | 0.06 | 0.03 | 0.55 | 0.05 | 0.09 | 0.06 | 0.09 | 0.08 |  | 0.05 | 0.04 | 0.07 | 0.07 | 0.10 |  | 0.20 | 0.50 |
| Expected | 1.07 | 2.76 | 0.87 | 34.9 | 0.07 | 0.10 | 2.84 | 5.07 | 0.79 |  | 0.34 | 0.18 | 1.13 | 0.05 | 0.19 |  | 11.4 | 36.7 |
| Absolute error | -0.08 | -0.20 | -0.10 | -1.88 | -0.05 | -0.73 | 0.00 | -0.14 | 0.07 |  | -0.03 | -0.01 | 0.01 | -0.01 | 0.02 |  | 0.58 | 1.76 |
| **Corning D** |  |  |  |  |  |  |  |  |  |  |  |  |  |  |  |  |  |  |
| Mean | 1.30 | 3.92 | 5.08 | 55.1 | 3.99 | 0.33 | 11.6 | 14.9 | 0.41 | 0.56 | 0.48 | 0.02 | 0.38 | 0.10 | 0.32 | 1.17 | 0.32 | 0.26 |
| sd | 0.04 | 0.08 | 0.07 | 0.84 | 0.11 | 0.05 | 0.20 | 0.23 | 0.04 | 0.03 | 0.04 | 0.04 | 0.04 | 0.05 | 0.10 | 0.11 | 0.06 | 0.06 |
| Expected | 1.20 | 3.94 | 5.30 | 55.2 | 3.93 | 0.23 | 11.3 | 14.8 | 0.38 | 0.55 | 0.52 | 0.02 | 0.38 | 0.10 | 0.10 | 0.97 | 0.29 | 0.24 |
| Absolute error | 0.10 | -0.02 | -0.22 | -0.18 | 0.06 | 0.10 | 0.29 | 0.12 | 0.03 | 0.01 | -0.04 | -0.01 | 0.00 | 0.00 | 0.22 | 0.20 | 0.02 | 0.02 |
